# Emotional resonance in the digital society: narrative mediation, platform hierarchy, and virtual consumption in cross-cultural sports communication

**DOI:** 10.3389/fpsyg.2026.1862323

**Published:** 2026-08-05

**Authors:** Junmian Wang, Guojun Liao

**Affiliations:** School of Physical Education, Yan’an University, Yan'an, China

**Keywords:** cross-cultural communication, digital social interaction, emotional contagion, sports live streaming, virtual consumption

## Abstract

This study examines digital emotional resonance in cross-cultural sports livestreaming through the analytical chain of host narrative–audience affective response–subsequent visible participation. Using the Chinese livestream of the Hakone Ekiden as an observational single-case study, we analyzed 97,878 publicly available interaction records collected across two race days. Sentiment scores for both audience comments and host commentary were generated using a fine-tuned BERT-base-Chinese model, and the main statistical analyses were conducted with 20-s time windows. Lagged correlation and impulse-response analyses were used to trace the temporal relationship between host and audience emotions; event-window models compared the differential roles of A1 technical commentary and A2 cultural-translation narrative; user-level grouping was used to examine platform-visible hierarchy; and forward-looking gifting-window models tested whether emotional alignment extended into subsequent gifting behavior. The results show that the pooled sample does not support stable zero-lag synchrony. Instead, the study provides weak and observational evidence of short-lag coupling, with the positive association appearing mainly when host emotion leads by approximately 40 s. A2 narrative does not consistently shorten audience response latency, but on the second race day it is associated with higher emotional maintenance during the 20–60 s after the event. Higher-level users show clearer same-window coupling and a higher probability of gift entry, although this result should be interpreted cautiously because user level may also reflect prior platform activity, platform tenure, and consumption tendency. Tighter emotional alignment between the host and the audience is associated with higher gift quantity and gift value in the subsequent time window. Overall, digital emotional resonance in this case is better understood as a conditional social process rather than as continuous, strong, and generalizable immediate synchrony.

## 1 Introduction

In recent years, as emerging media have continued to expand, a growing body of research has begun to incorporate a wide range of digital social activities into scholarly analysis (Chi et al., 2025; Lee and Theokary, 2021). Yet sports, despite being one of the fields most deeply entangled with digital culture, has not received a level of academic attention commensurate with its social significance. In practice, both the circulation of sports content and the forms of audience participation and emotional investment have already been reorganized into a new interactive order within platform environments. Put differently, in the digital society, sports livestreaming is no longer merely an online extension of traditional television broadcasting; it has become an interactive communal space continuously shaped by platform mechanisms (Gao et al., 2024). For example, when elements such as host commentary, audience comments, user levels, fan badges, and virtual gifts are presented simultaneously on the same interface, emotion is no longer merely an individual response attached to the atmosphere of the competition, but becomes a digital social process organized by narrative, amplified by the crowd, and shaped by platform mechanisms (Pan et al., 2021). What is truly worth asking is no longer simply whether viewers generate emotion, but how these emotions are interpreted, shared, made visible, and further transformed into collective action within virtual communities (Pan et al., 2021). This point becomes even more evident in the context of cross-cultural sports live streaming. For domestic Japanese viewers, the Hakone Ekiden itself carries a strong tradition of distance running culture, memories of intercollegiate rivalry, and annual ritual significance; but for culturally different audiences watching the broadcast on livestreaming platforms in another country, the images and commentary of the same event are not naturally transparent. Viewers can certainly see moments such as relay handoffs, slowing pace, overtaking, and sprint finishes, yet they may not immediately understand why a particular section matters, why a particular university carries additional emotional weight, or why a runner’s change in rhythm should be read as a tactical signal rather than a random fluctuation. In such a communicative context, the host is no longer merely a broadcaster of the event, but also a translator of cultural meaning: he must transform a sporting object embedded in a foreign context and its race situation into a public event that audiences on a cross-cultural platform can enter, understand, and remain willing to follow.

Existing studies have accumulated a considerable amount of discussion on live-streaming interaction, emotional contagion, and platform participation, but there are still two obvious shortcomings in explaining the mechanisms of emotion generation in cross-cultural sports live streaming (Li et al., 2024; Qian and Seifried, 2023). First, prior research has mostly adopted relatively static analytical approaches and has tended to understand audience interaction and consumption as relatively stable responses to content value, platform experience, or satisfaction, while paying comparatively little attention to short-term emotional fluctuations, lagged coupling, and behavioral amplification triggered by real-time narrative (Qian and Seifried, 2023; Shao and Huang, 2025). Second, although some studies have recognized the synchronous and interactive features of livestreaming, few have integrated host narrative, audience emotion, platform hierarchy, and gifting behavior within the same temporal mechanism chain. As a result, a more consequential question remains insufficiently answered: in cross-cultural sports livestreaming, does hosts’ interpretive narrative significantly shape audience emotional contagion, and does it further amplify interaction and consumption behavior? Against this backdrop, the present study takes the Chinese livestream broadcast of the Hakone Ekiden as its empirical case and examines it through a mechanism chain linking narrative, emotion, and interaction/consumption. Unlike previous research that has treated commentary primarily as a channel of information delivery, this study focuses on how hosts engage in ongoing cultural translation through discursive practices such as historical contextualization, background explanation, assessments of competitive dynamics, and strategic interpretation. Audience emotion is therefore conceptualized as a collective affective process that accumulates, fluctuates, and becomes publicly visible through livestream interaction, while gift-giving is understood as the visible platform-based expression of that process.

Following this line of thinking, this article attempts to place host narrative, audience emotion, user hierarchy, and gift-giving behavior across the two race days of the Hakone Ekiden on the same timeline for analysis. On the one hand, all formal tests in the article are uniformly based on the main 20-s time window in order to preserve as much detail as possible regarding short-term fluctuations, lag changes, and emotional transformation processes in livestream interaction. On the other hand, the host’s commentary is transcribed into text and further divided into two types, technical reporting and cultural translation narrative (A1/A2), in order to compare their different roles in emotional generation. Based on this design, this article seeks to further reveal the mechanisms of digital social interaction in cross-cultural sports communication, and to test whether digital emotional resonance between hosts and audiences is manifested as neat and uniform immediate synchrony or as a form of dynamic coupling with lag, and whether such coupling will continue to be amplified under the joint action of the livestreaming platform and gifting mechanism and be transformed into visible behavioral presentation. These findings not only help digital marketing practitioners and cross-cultural broadcasting platforms more accurately grasp narrative direction, the pathways of emotional resonance, and their stage-specific features in sports-cultural communication, but also help them organize commentary, interaction prompts, and platform mechanisms more effectively at key moments of communication, thereby enhancing audience emotional involvement, community cohesion, and sustained participation.

## 2 Literature review and research hypotheses

### 2.1 Digital emotional contagion and resonance

With the rapid development of the internet, studies in recent years have shifted emotional contagion from the narrow sense of offline face-to-face imitation increasingly toward interactive contexts in digital media. Existing studies have pointed out that digital emotional contagion is not an accidental phenomenon of emotional attachment, but a process that is triggered in online interaction, diffused, and further amplified by platforms (Goldenberg and Gross, 2020; Li et al., 2025b). Some studies have further summarized it as several interrelated conditions, including whether interaction itself is sustained, whether emotional transmission has directionality, and whether the interacting parties can form some kind of similar perception (Bai et al., 2025; Herrando and Constantinides, 2021). In other words, in digital environments, emotion should be understood more as a process that is gradually generated and continuously flows in relational interaction, rather than as a static and unchanging psychological outcome inside the individual. This point is especially critical in livestreaming contexts. Unlike general asynchronous media, livestreaming does not first produce content and then wait for audiences to respond afterward, but continuously layers expression, response, follow-up, and feedback together within the same temporal interface (Luo et al., 2026; Muñoz, 2026). What the host says, how viewers respond, how comments accumulate, and how emotion is further amplified often all occur within extremely short intervals of time. Existing studies have already pointed out that social platforms themselves have the capacity to amplify emotional diffusion (Goldenberg and Willer, 2023; Schöne et al., 2026); in tourism livestreaming contexts, emotional coupling between streamers and viewers has also been shown not to be a one-way stimulus–response relationship, but rather a dynamic process of real-time progression and bidirectional feedback (Chi et al., 2025). Therefore, so-called “resonance” does not necessarily have to appear as a strict overlap between two actors at the same moment; it may also be accompanied by brief lag, rhythmic misalignment, and subsequent echo.

In this study, digital emotional resonance is not understood as complete emotional synchrony between the host and the audience at the same moment. Livestreaming interaction has a continuously unfolding temporal structure: host commentary first introduces event information, emotional cues, and interpretive frames into the public interface, while the audience gradually forms a visible collective affective surface through reception, interpretation, and comment-based response. Accordingly, this study defines emotional contagion as the diffusion of emotional expression in mediated interaction, and defines emotional resonance as an interpretable temporal relationship between host commentary emotion and audience comment emotion across consecutive time windows. By contrast, synchrony refers only to co-movement within the same window, whereas emotional alignment refers to the closeness of the two sentiment scores within the same window. Based on this distinction, the study does not assume that resonance must necessarily appear as immediate synchrony. Instead, it further examines whether same-window association, short-lag response, and subsequent echo together constitute an identifiable pattern of temporal coupling. Based on the above theoretical reasoning, the following hypothesis is proposed:

*H1:* In cross-cultural sports livestreaming, host commentary emotion and audience comment emotion show an identifiable temporal coupling relationship within the same time window or short-lag windows.

### 2.2 Narrative mediation and cross-cultural translation

Different from simply discussing emotional synchrony, another line of research pays more attention to what kind of discursive organization hosts use in a highly immediate media context like livestreaming to transform constantly flowing information into an interpretive framework that audiences can enter (Cao et al., 2025). Existing studies have pointed out that livestreaming differs from general online communication not merely because it is “happening now,” but because it simultaneously possesses features such as immediate response, co-presence, and continuous interaction (Deng et al., 2021). As this synchronous interaction is repeatedly reproduced, originally short-term contact relations may gradually settle into more stable parasocial ties (Deng et al., 2022). At the same time, some studies understand livestreaming as a continuous process of value construction (Xie et al., 2022), and also emphasize that livestreaming is transforming previously passive viewing activities into an interactive process of multi-layered involvement (Hua et al., 2023). In this sense, streamers or hosts are not merely people who speak out information, but organizers of the entire order of interaction and the rhythm of understanding. Taking one step further, how hosts “speak” itself directly affects subsequent interactive outcomes. Existing studies have found that the emotional valence in streamers’ language affects livestream performance (Ma et al., 2024), and different topic types also lead to different forms of user participation (Luo et al., 2024). In the context of cross-cultural sports communication, this becomes even more evident. Research on athletes’ social media presentation during the Tokyo Olympics shows that nationalism, gender, and platform environment jointly shape the expression of digital sports narratives (Xu et al., 2024); another study on the Beijing Winter Olympics also shows that even on the same platform, there are still clear differences between official media framing and public ways of understanding (Yang et al., 2023). To some extent, this indicates that cross-cultural sports communication has never been a matter of moving an event over “as it is,” but a process of ongoing selection, interpretation, translation, and re-narration.

Based on the above discussion, this study further distinguishes between two types of host narrative. The first is closer to the technical transmission of immediate race conditions, rank changes, and race progression; the second is oriented toward audiences who are not naturally familiar with the cultural background of the event, and it explains and translates event meaning, athlete situations, school traditions, and competitive dynamics. The difference between A1 and A2 lies not simply in the length of expression or the strength of emotion, but in whether the host provides audiences with a meaning frame for understanding why a particular race segment matters. From this perspective, the role of A2 cultural-translation narrative may not necessarily appear as an immediate increase in audience emotion, nor does it necessarily shorten the audience’s initial response latency. Rather, it is more likely to be reflected in differences in the rhythm through which audience emotion enters, persists, or recedes after the narrative event. Therefore, this study uses the latency to the audience emotional peak and the level of emotional maintenance during the 20–60 s after the event as two pre-specified indicators to examine whether A2 corresponds to different post-event audience affective trajectories. The following hypothesis is proposed:

*H2:* Compared with A1 technical narrative, A2 cultural-translation narrative is associated with differences in post-event audience affective trajectories, especially in response latency or short-term emotional maintenance.

### 2.3 Parasocial participation and platform stratification

Online sports spectatorship is not simply the addition of many individual reactions, but continuously generates a form of public emotion that can be jointly perceived through sustained interaction. Existing studies, from the perspective of interaction ritual chains, point out that online spectatorship can also produce collective emotional energy, and that this energy comes not only from the competition itself, but also from shared attention, synchronized response, and mutual confirmation among viewers at the same moment (Zhang et al., 2024). At the same time, some studies have found that digital sports participation is closely related to the psychology of “fear of missing out,” which largely explains why real-time immediacy is so important in sports livestreaming: once a key moment occurs, viewers not only want to “know the result,” but also hope that they have not missed the moment that is being collectively experienced (Lee and Na, 2024). In other words, emotion in sports livestreaming does not remain an immediate reaction inside the individual, but can easily be transformed into a collective experience of presence through synchronized interaction.

Further, this public emotion does not exist in the abstract, but often attaches itself to those continuously visible forms of interaction in the livestream room. Research on bullet comments in interviews with female athletes has already shown that danmu is not fragmented commentary attached beside content, but itself a parasocial emotional surface that can be jointly seen and jointly continued (Zhang, 2026). This kind of digital sports emotion does not remain at the level of expression, but may further spill over into clearer and more action-oriented forms of participation (Chen and Kwak, 2025). This indicates that in sports livestreaming contexts, comments, danmu, symbolic responses, and even subsequent behavior are not several separate links, but together constitute a continuous process through which emotion moves from generation and diffusion to visible manifestation. From this perspective, the sports livestream room is not merely a channel for watching an event, but more like a stratified public space jointly organized by host commentary, audience interaction, platform prompts, identity markers, and visible feedback. Although different viewers share the same match, their degree of visibility, capacity for interaction, and mode of action within this space are not entirely the same. Research on global sports event communication also reminds us that once any sporting event enters different media systems, it does not circulate directly with a single and fixed meaning, but is reinterpreted within different platform environments and communicative frames (Abdul Rehman and Jebril, 2025). For the Chinese broadcast of the Hakone Ekiden, this is equally important: it is not only an entry point for watching a foreign event, but also a site where emotional sharing, interactional clustering, and identity differentiation continue to occur after cultural narrative translation.

Precisely for this reason, public emotion in the livestream room does not unfold in a completely homogeneous space. Users who occupy more visible positions and are more likely to be noticed by others are often more likely to enter this emotional circuit earlier and further transform emotion into clearer interactive behavior. Based on the above theoretical reasoning, we propose the following hypothesis to test whether platform stratification influences how viewers receive hosts’ emotional signals and their subsequent visible participation:

*H3:* Compared with low-level users, high-level users are more likely to show visible coupling associated with host emotion and to enter the gifting layer.

### 2.4 Emotional manifestation and virtual consumption behavior

In sports livestreaming contexts, emotion does not remain at the level of comments and danmu, but further transforms into behavioral forms that are more visible and more costly. Existing studies have shown that emotional experience in livestreaming not only affects user participation, but also further brings actual economic returns (Lin et al., 2021); and once such participation enters a process of sustained feedback, it is continuously accumulated and reinforced in interaction (Song et al., 2025). For this reason, gifting behavior cannot be simply regarded as an incidental consumption behavior on the platform; it is more like a mode of expression with clear motivation and social meaning (Gong et al., 2023). Correspondingly, some studies have also found that emotional contagion in mediated environments further promotes the formation of subsequent behavioral intentions (Robin Tang et al., 2024; Yu L. et al., 2025). This indicates that on livestreaming platforms, emotion does not simply end once it has been “expressed,” but often continues along the interaction chain and, at certain moments, transforms into clearer action outcomes (Yu T. et al., 2025).

For this article, this point is especially important. Compared with ordinary comments, gifting behavior has stronger visibility, publicity, and symbolism. It not only means that viewers are willing to pay a cost for their current emotional experience, but also means that this emotion has been transformed from an inner feeling into an action signal that can be seen by others in the livestream room (Meng et al., 2021; Wang et al., 2025). In other words, the livestreaming platform is not a homogeneous interactive space, but a stratified field that further organizes emotion, identity, and visible participation. Under such a mechanism, the tighter the emotional alignment between hosts and audiences, the more likely viewers are to move beyond ordinary interaction and enter more explicit gifting expression and consumption behavior. On this basis, to test whether emotional alignment between hosts and audiences further promotes the occurrence of subsequent gifting behavior and changes in its intensity, this study proposes the following hypothesis:

*H4:* The tighter the emotional alignment between the host and the audience, the higher the gift quantity and gift value in the subsequent window.

## 3 Materials and methods

### 3.1 Data collection

This study takes the real-time Chinese-language rebroadcast of the Hakone Ekiden on Chinese platforms on January 2 and 3, 2026, as its research object, and selects the most popular livestream room on Douyin during the race period as the observational case. The research team used a Python-based web crawler to collect publicly visible interaction records from the two race days. After manual cleaning and field standardization, 42,425 valid records were retained for Day 1 and 55,453 for Day 2, yielding a total of 97,878 valid interaction records (see Table 1). At the 20-s window level, Day 1 produced 1,008 windows and Day 2 produced 1,028 windows; among these, 997 and 1,016 windows, respectively, contained both audience and host sentiment measurements, while 441 and 546 windows, respectively, contained at least one gifting event. This study is a low-risk observational study based on publicly available digital trace data. It uses only publicly visible livestream interaction records, does not access private profiles, private messages, or any non-public materials, and does not involve direct intervention, experimental manipulation, recruitment of participants, or direct contact with individual users. The original logs contained platform-displayed usernames and related fields, but these fields were used only for data cleaning and record verification. They were not used to identify, contact, track, or profile any specific user. In the manuscript, results, and any publicly available materials, all usernames and other potentially identifiable information have been removed or de-identified, and the results are reported only in aggregated form. Given the low-risk nature of the study and the fact that no personally identifiable information is disclosed in any public materials, the study has been confirmed as exempt from full ethics review, and individual informed consent was not required.

**Table 1:** Descriptive statistics of the two race days.

Indicator	Day 1	Day 2
Valid interaction records	42,425	55,453
Host-commentary rows	6,219	6,813
Gift rows	1,100	1,621
Gift-value sum (CNY)	1887.5	3167.5
Comment sentiment mean	0.1037	0.1046
Host sentiment mean	0.0699	0.0657
A1-coded host rows	4,463	4,859
A2-coded host rows	1,361	1,349

This case was selected, on the one hand, because the Hakone Ekiden itself has distinctive cross-cultural communication attributes; on the other hand, host commentary, audience comments, level markers, and gifting mechanisms coexist in the Chinese livestream room, making it possible to present relatively completely the unfolding process of the mechanism chain of “narrative–emotion–interaction/consumption.” In other words, this setting contains both observable emotional flows and visible behaviors that can be directly recorded, while also providing the culturally translational narrative interaction that this study is concerned with.

### 3.2 Data preprocessing and sentiment measurement

Before the formal analysis, this study first cleaned and organized the raw data in a unified manner, and aligned the audience comment texts and host commentary texts according to real-time timestamps so that different information streams could be compared within the same temporal framework. For sentiment measurement, this study used a BERT-base-Chinese model fine-tuned on 361,744 labeled Sina Weibo posts to calculate continuous sentiment weights for both audience comments and host commentary. The source corpus contains four emotion labels: 199,496 joy texts, 51,714 anger texts, 55,267 disgust texts, and 55,267 low-mood texts. After training, the model achieved an overall accuracy of 0.854, precision of 0.862, and F1 score of 0.883 on the source-corpus validation set. It should be noted that the validation results from the source corpus cannot be directly equated with the model’s full validity in the context of Douyin sports livestreaming. Weibo texts and Douyin sports livestream comments may differ in abbreviations, irony, sports terminology, emojis, platform-specific expressions, and emotional intensity. Therefore, this study treats sentiment scores as continuously traceable indicators of textual emotional expression in the livestream room, rather than as direct inferences of individuals’ internal psychological states. Accordingly, the subsequent results are interpreted as patterns of textual emotional expression rather than direct evidence of individual psychological emotion. To preserve the dynamic temporal details of the interaction process as much as possible, this study conducted all formal statistical analyses using 20-s time windows, denoted as t, and aggregated audience comment emotion and host commentary emotion separately by calculating window-level means, as follows Equations 1, 2:


$$\bar{A}t=\frac{1}{n_{t}^{A}}\sum i\in tA_{i}$$ (1)

$$\bar{H}t=\frac{1}{n_{t}^{H}}\sum j\in tH_{j}$$ (2)

Here, $\bar{A}t$ represents the average audience emotion in the t -th 20-s time window, and $\bar{H}t$ represents the average host emotion in the same window. $A_{i}$ and $H_{j}$ correspond, respectively, to the sentiment weights of comment texts and host texts. This treatment, on the one hand, can preserve short-term emotional fluctuations in livestream interaction, and on the other hand, also helps compare the emotional coupling relationship between hosts and audiences on a unified time scale.

### 3.3 Core variables and coding procedures

For the narrative variable, this study classified host commentary texts using an A1/A2 coding scheme. The coding process first used rule-based cues for assisted identification and then conducted manual review in context, because the distinction between technical reporting and cultural-translation narrative depends not only on keywords, but more importantly on the function of the utterance within the livestream narrative. A1 mainly refers to technical narrative, including race-condition reporting, rank changes, pace judgment, chasing, and relay handoffs. A2 refers to cultural-translation content, including historical background, institutional context, athlete identity, competitive structure, strategic analysis, and pressure interpretation. To assess coding reliability, we invited three independent coders to conduct manual review. Two coders independently coded the sampled texts, and a third coder adjudicated disagreements or low-confidence cases. The independent coding results showed an intercoder agreement rate of 81.7%, with Cohen’s *κ* = 0.714, indicating an acceptable level of consistency for the A1/A2 classification. Utterances such as greetings, transitions, platform interactions, discourse particles, or semantically incomplete fragments that could not be stably classified as either A1 or A2 were treated as unclassifiable and were not included in the H2 narrative event-window analysis.

For the stratification variable, this study retained user-level information and treated it as an observational marker of platform-visible hierarchy in order to compare differences between high-level and low-level users. This treatment is based on the fact that the livestream room is not a flat space: users at different levels may differ in visibility, modes of participation, and the likelihood of entering the gifting layer. At the same time, this study does not treat user level as an exogenous causal variable. User level may also capture prior consumption, platform tenure, baseline activity, fan identity, and repeated participation; therefore, it is more appropriately interpreted as an observational marker of stratified participation.

For the behavioral variables, this study mainly focuses on gift quantity and gift value. The former is used to describe the occurrence and increase of gifting behavior, while the latter is used to measure whether this behavior involves higher-intensity value output. To further capture the emotional proximity between the host and the audience within the same window, this study calculates an emotional alignment indicator, defined as the opposite of the emotional distance between host and audience (Equation 3):


$$\begin{array}{c} \text{Alig}n_{t}=-|{\bar{A}}_{t}-{\bar{H}}_{t}| \end{array}$$ (3)

Here, ${\bar{A}}_{t}$ denotes the average audience comment sentiment score in the t -th 20-s window, and ${\bar{H}}_{t}$ denotes the average host commentary sentiment score in the same window. A higher value of this indicator indicates a smaller emotional distance between the host and the audience, and therefore a higher degree of emotional alignment.

### 3.4 Analytical strategy and testing procedures

In the empirical analysis, this study does not compress all research questions into a single statistical method. Instead, it follows the mechanism chain of narrative–emotion–interaction/consumption and matches different levels of research questions with corresponding analytical procedures. The 20-s window is used as the main temporal scale throughout the study. This window was selected because it provides a feasible compromise between temporal sensitivity and measurement stability: on the one hand, 20 s is short enough to preserve the short-term response rhythm of livestreaming interaction; on the other hand, it reduces the instability of sentiment means caused by too few comments in shorter windows. For empty windows without corresponding text records, sentiment means were not calculated. Low-comment windows that still contained valid interactions were retained, but interpreted cautiously; where appropriate, comment volume and host-commentary volume were controlled in the relevant models. Windows lacking either host or audience sentiment measurements were excluded from the main analyses that required paired host–audience sentiment. First, to test H1, this study uses same-window correlation, lagged correlation, and 1-s impulse-response analysis to examine whether the host commentary emotion series and the audience comment emotion series show same-window or short-lag associations. The basic form of the lagged-correlation function is as follows Equation 4:


$$\begin{array}{c} r(\ell )=\text{corr}(\bar{H}t,\bar{A}t+\ell ) \end{array}$$ (4)

Here, ℓ denotes the lag order, with each unit corresponding to one 20-s time window; when ℓ>0 , host emotion leads audience emotion. The purpose of this specification is not merely to test whether the two series move simultaneously, but to determine whether resonance in the livestream room is better understood as immediate synchrony or as a lagged form of dynamic coupling.

Further, turning to host narrative itself, the study identifies narrative event windows on a 20-s basis and compares A1 and A2 commentary on the same temporal scale. The analysis focuses primarily on the lag to peak audience emotion and the short-term maintenance level during the 20–60 s following an event, in order to assess whether culturally translational narrative alters the pathway through which audiences enter emotional resonance. The corresponding regression specification is given below Equation 5:


$$\begin{array}{c} Y_{i}=\alpha +\mathrm{\beta A}2_{i}+\text{γBaselin}e_{i}+\delta {\bar{H}}_{i}+\varepsilon_{i} \end{array}$$ (5)

Here, $Y_{i}$ denotes either post-event peak latency or the level of emotional maintenance during the 20–60 s following an event; $A2_{i}$ indicates whether a given event window belongs to the culturally translational narrative category; $\text{Baselin}e_{i}$ captures pre-event baseline emotion; and ${\bar{H}}_{i}$ represents mean host emotion within that window. The purpose of this specification is to determine whether A2 alters the temporal pathway through which audiences enter emotional resonance, rather than merely testing whether it directly elevates average emotion.

Building on this step, this study further incorporates platform hierarchy and gifting behavior into the same temporal chain of analysis. High-level and low-level users are compared within the same windows to examine differences in emotional coupling, response rhythm, and gift entry. The gifting models are also specified according to the nature of the outcome variables: gift quantity is a count outcome, while gift value is right-skewed and contains many zero-valued windows. Therefore, the forward-looking linear model is treated as a transparent baseline association model, supplemented by robustness checks using count models and two-part models. Where feasible, the models also control for prior-window gifting, comment volume, host-commentary volume, day fixed effects, race stage, and major race-event shocks. The corresponding forward-looking model is specified as follows Equation 6:


$$\begin{array}{c} Y_{t+1}=\alpha +\text{βAlig}n_{t}+\gamma X_{t}+\varepsilon_{t} \end{array}$$ (6)

Here, $Y_{t+1}$ represents the gift quantity and the log-transformed gift value in the next 20-s time window, $\text{Alig}n_{t}$ represents the degree of emotional alignment in the current window, and $X_{t}$ represents the contemporaneous control variables. In other words, the real question we want to answer here is: when the emotional relationship in the livestream room becomes tighter and more concentrated, is it more likely to be externalized as monetized supportive behavior.

Overall, this study first conducts tests based on the pooled sample across the 2 days, and then uses the day-specific results for Day 1 and Day 2 as auxiliary comparisons. On the one hand, this approach can prevent single-day fluctuations from exerting overly strong influence on the conclusions; on the other hand, it also preserves, as much as possible, the differences between the two race days. We adopt this arrangement not to make the method look more complicated, but to place host narrative, audience emotion, platform hierarchy, and gifting behavior back into one continuously unfolding process for deeper understanding and analysis.

## 4 Results

### 4.1 Process display and overall trends

**Figure 1:**
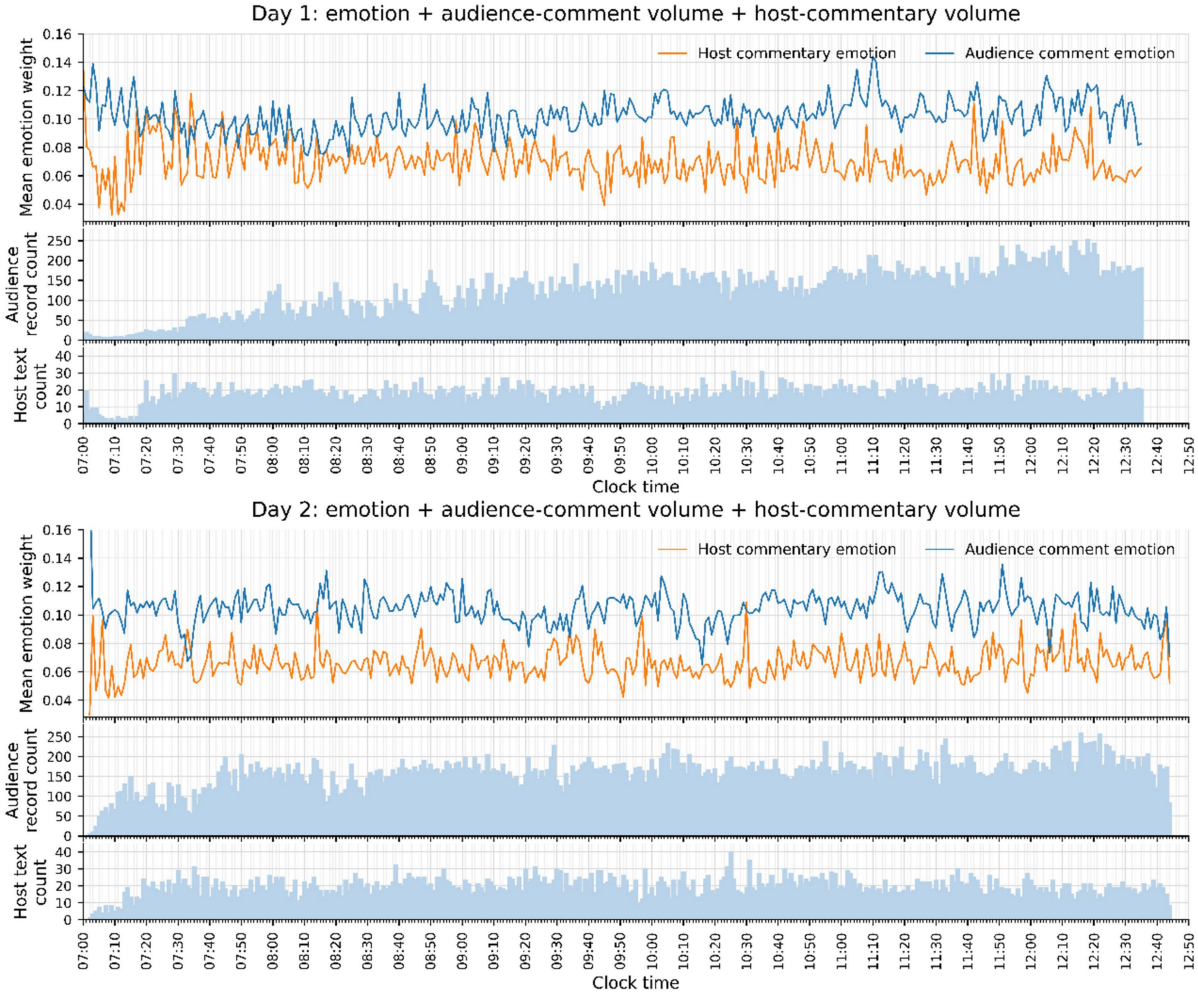
Two-day minute-level complete process chart.

Figure 1 displays the overall fluctuation of the two livestream days at the 1-min level. In general, audience comment emotion remains consistently higher than host commentary emotion, but the two series do not move in perfectly matched rhythms. On the first race day, the host and audience emotion curves show relatively similar movement during several key periods, yielding a pattern closer to weak synchrony overall. On the second race day, by contrast, it is more common to observe host emotion moving first and the comment layer following afterward, indicating that the interactional rhythm of the Chinese Hakone Ekiden rebroadcast differs across race days. At the same time, variation in comment volume and host-commentary volume does not mechanically mirror the emotional curves: In some intervals, interaction density increases without a corresponding strengthening of emotional alignment, whereas in others, host emotion shifts first and audience response emerges only with a delay. Taken together, the minute-level figure is better suited to presenting the overall contour of fluctuation across the two race days than to serving as a basis for formal identification. For this reason, the more analytically informative judgments are made at the 20-s window level, where the temporal relationships among host narrative, audience emotion, platform stratification, and gifting behavior can be examined in finer detail.

### 4.2 Emotional resonance: synchrony does not always occur at the same moment

**Figure 2:**
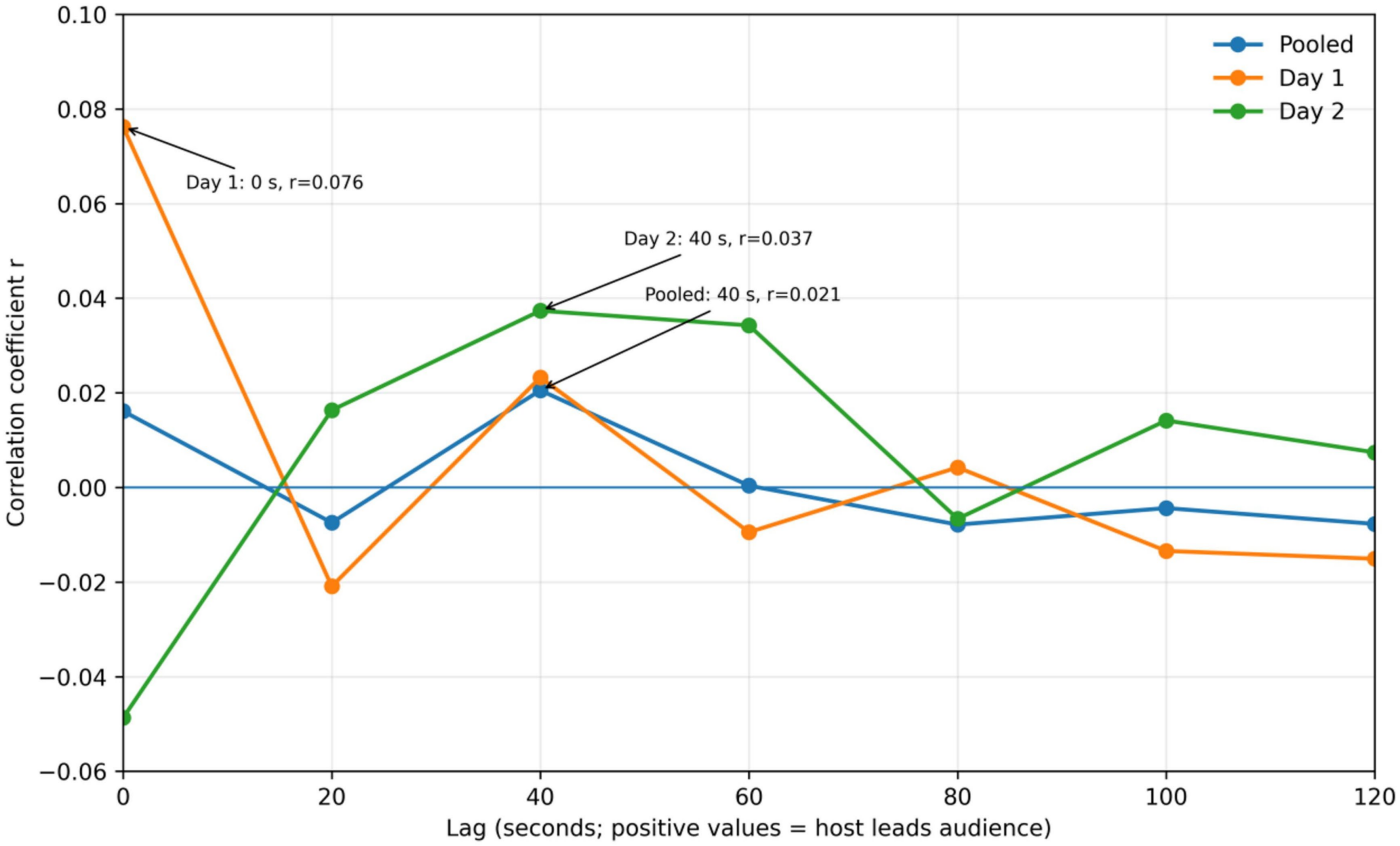
Lag-correlation test results of the host’s and audience’s emotions (20-s window).

In the pooled sample, once the analysis was conducted at the 20-s window level, no stable zero-lag synchrony was observed between hosts and audiences. The same-window correlation is only *r* = 0.021 (*p* = 0.355), with a 95% confidence interval of [−0.023, 0.064]. On this criterion alone, there is insufficient evidence to claim significant immediate resonance between hosts and audiences. Once short temporal lags are taken into account, however, the positive association becomes much clearer: in the pooled sample, the positive peak appears primarily at a short lag of approximately 40 s. This suggests that emotion in the livestream room is not always transmitted at the very moment the host speaks. More often, it appears to enter the audience comment layer only after the host’s commentary has been processed over a brief interval. The contrast becomes even clearer when the two race days are examined separately. On the first race day, the same-window correlation is modest but statistically significant, and the strongest positive correlation also occurs at zero lag, indicating a pattern closer to weak synchrony or near-immediate coupling. The second race day looks markedly different: the same-window correlation is negative and non-significant (*r* = −0.021, *p* = 0.101), yet once host emotion is allowed to lead, the strongest positive correlation appears at a short lag of about 40 s (*r* = 0.037, *p* = 0.015). This indicates that resonance is not absent on Day 2; rather, it takes the form of a delayed structure in which host interpretation comes first and audience response follows. The lag-correlation trajectories shown in Figure 2 are consistent with this pattern: the Day 1 peak lies closer to zero lag, whereas the positive peaks in the pooled sample and on Day 2 are concentrated more in the short-lag range than in the same-window position.

**Figure 3:**
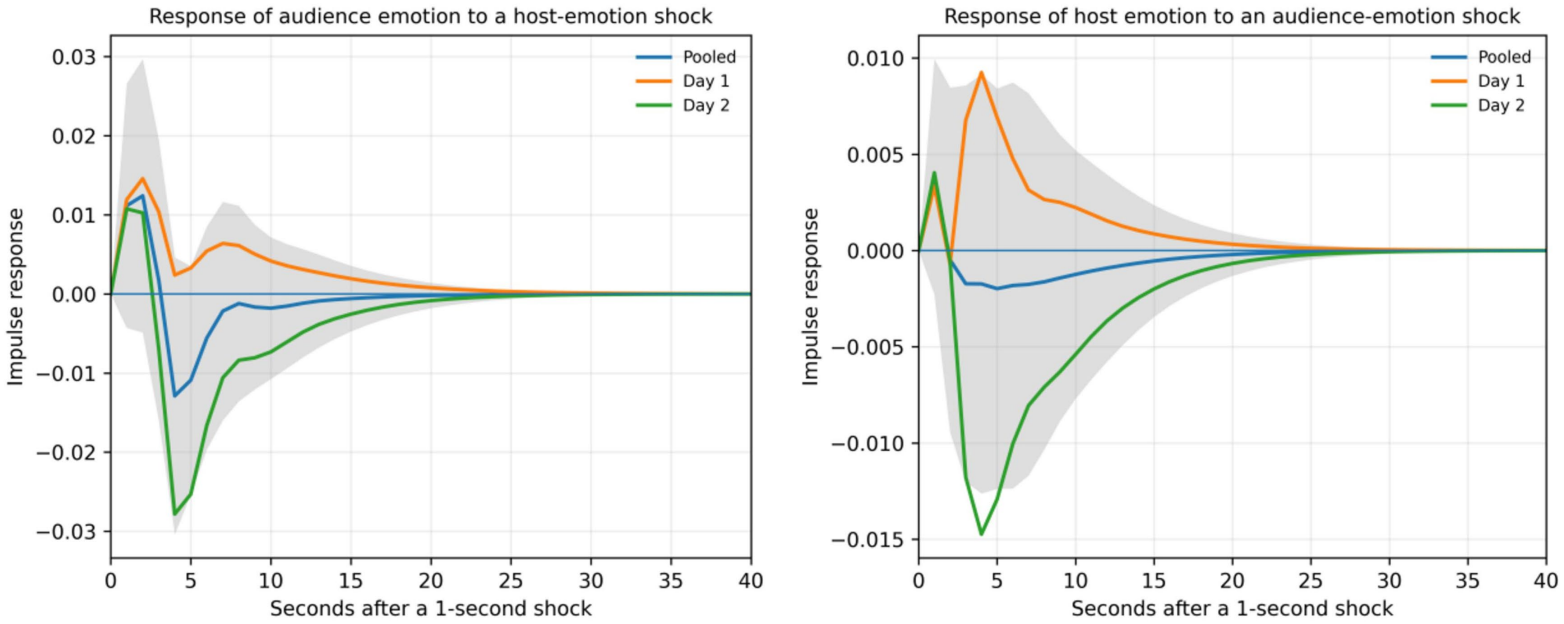
Impulse response function based on 1-s interval VAR.

To further determine whether this relationship exists only at the 20-s aggregation level, this study conducted an auxiliary test on the 1-s time series. The results show that the pooled sample exhibits a significant positive correlation around 18 s (*r* = 0.027, *p* = 0.035); the first race day is already significant at zero lag (*r* = 0.036, *p* = 0.006); and the second race day shows a locally significant positive peak within the short-lag interval of 25–41 s. Consistent with this result, the 1-s VAR impulse-response function shown in Figure 3 further indicates that the effect of host emotional shocks on audience emotion does not remain at the contemporaneous second, but continues to unfold over the following several tens of seconds; by contrast, the reverse shock from audience emotion to host emotion is shorter and more unstable. In other words, the 1-s results do not change the direction of the main 20-s test, but further unfold this short-lag relationship on a finer temporal scale.

Taken together, the main 20-s window analysis, the 1-s auxiliary test, and the supplementary time-series diagnostics suggest that H1 is better characterized as receiving limited partial support. The supplementary diagnostics show that the relevant sentiment series passed the ADF stationarity tests, but the raw sequences still exhibited significant autocorrelation. After pre-whitening and Benjamini–Hochberg correction across multiple lags, the evidence from the 20-s lagged correlations became substantially weaker. In the 1-s VAR auxiliary analysis, BIC selected a 14-lag specification. Residual diagnostics were generally acceptable in the day-specific models, whereas the pooled model still showed some residual autocorrelation. Therefore, the emotional resonance identified in this study should not be understood as a stable, strong, or causal synchrony mechanism. It is more appropriately interpreted as a weak, local, and observational pattern of temporal coupling. The raw lagged correlations and graphical results suggest that Day 1 is closer to weak near-immediate coupling, whereas Day 2 may be closer to a short-lag structure in which host commentary emotion precedes and audience comment emotion follows. However, this pattern is not robust after accounting for autocorrelation and multiple-testing correction. Accordingly, H1 receives only limited and conditional support.

### 4.3 Emotional regulation by culturally translational narrative

When only the pooled sample is considered, A2 does not operate like a strong stimulus that immediately elevates emotion. In the 20-s event-window model, A2 has no significant effect on peak latency (*β* = 1.048, SE = 0.793, 95% CI [−0.506, 2.602], *p* = 0.187), nor does it significantly increase emotional maintenance during the subsequent 20–60 s (*β* = 0.008, SE = 0.005, 95% CI [−0.002, 0.018], *p* = 0.126). This indicates that culturally translational narrative does not produce a uniform general effect in the pooled sample. Put differently, the presence of A2 does not, by itself, ensure that audiences enter emotion more quickly or that mean emotion rises immediately.

Once the two race days are examined separately, however, the contrast becomes much clearer. On the first race day, A2 shows no significant difference in either peak latency (*β* = 0.420, SE = 1.129, 95% CI [−1.793, 2.633], *p* = 0.710) or subsequent maintenance (*β* = −0.003, SE = 0.008, 95% CI [−0.019, 0.013], *p* = 0.668), and the raw maintenance means in A1-only and A2 windows are nearly identical (0.1019 vs. 0.1014). Under these conditions, A2 appears more as an interpretive supplement embedded within the overall rhythm of commentary than as a narrative form that draws a distinct emotional boundary on its own. The second race day, however, shows a different pattern. Although A2 still does not significantly shorten peak latency (*β* = 1.642, SE = 1.115, 95% CI [−0.543, 3.827], *p* = 0.141), it exerts a stable and clearly positive effect on emotional maintenance during the following 20–60 s (*β* = 0.019, SE = 0.007, 95% CI [0.005, 0.033], *p* = 0.006), and the raw means point in the same direction: 0.1032 for A1-only and 0.1051 for A2. In other words, the main effect of A2 here lies not in accelerating emotional response, but in slowing the rate at which emotion recedes.

**Figure 4:**
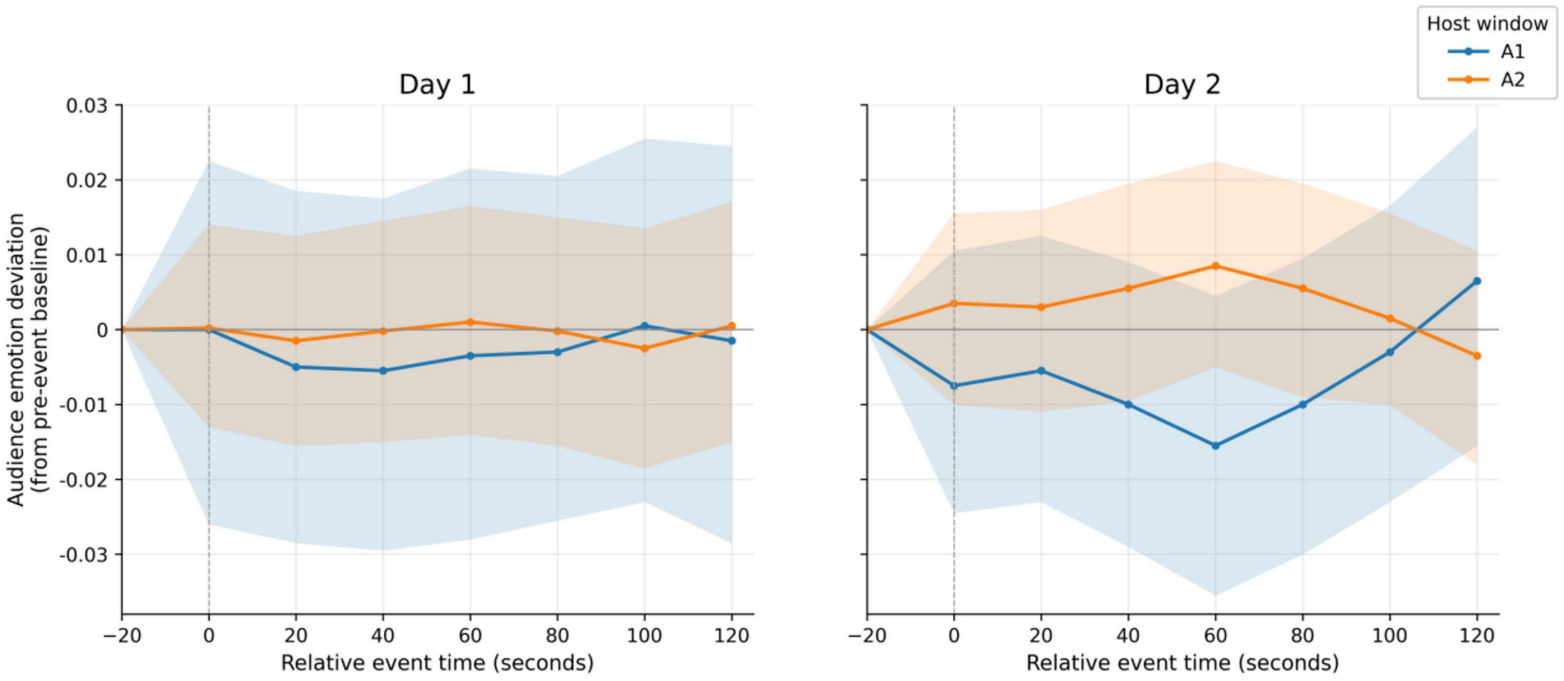
The research trajectory of events surrounding the A1 and A2 host windows.

This interpretation is also broadly consistent with the event-study trajectories shown in Figure 4. On Day 1, the post-event trajectories of A1 and A2 remain generally close to each other, with no stable separation between the two. On Day 2, however, the A2 trajectory stays more consistently above the A1 trajectory during the middle post-event windows. Overall, H2 receives conditional support. A2 does not consistently shorten the latency to the audience emotional peak in either the pooled sample or the day-specific models. The clearer evidence appears mainly on Day 2, where A2 is associated with higher audience comment emotion maintenance during the 20–60 s after the event. Therefore, cultural-translation narrative should not be understood as a narrative form that generally accelerates audiences’ emotional entry. In this case, it is more likely to correspond to the continuation of the post-event affective trajectory rather than to an earlier initial response. At the same time, this result also suggests that A2 may not be an internally homogeneous narrative category. Different types of cultural-translation content may have different effects on subsequent emotional maintenance, which should be further distinguished and tested in future research.

### 4.4 The predictive role of platform level

Based on the user-level distribution in the pooled sample, this study divides viewers into four quartile groups, with Q1 to Q4 corresponding to the lowest 25% through the highest 25% of the user-level range. The subgroup results show that level differences first appear in the visibility of emotional coupling. A significant same-window correlation is observed between high-level users’ comment emotion and host commentary emotion (*r* = 0.096, *p* < 0.001), whereas the low-level user subsequence does not show the same relationship (*r* = −0.004, *p* = 0.860). This suggests that, in this case, the host’s emotional signals do not enter all audience comment layers in the same way, but are more clearly reflected in the visible responses of high-level users. However, this result does not mean that platform level itself directly causes stronger emotional responses. User level may also capture differences in prior consumption, platform tenure, baseline activity, fan identity, and repeated participation. Therefore, this study treats user level more cautiously as an observational marker of stratified participation: it indicates that different user strata vary in the visible degree of emotional coupling, but it cannot by itself prove a causal effect of user level on emotional response.

**Figure 5:**
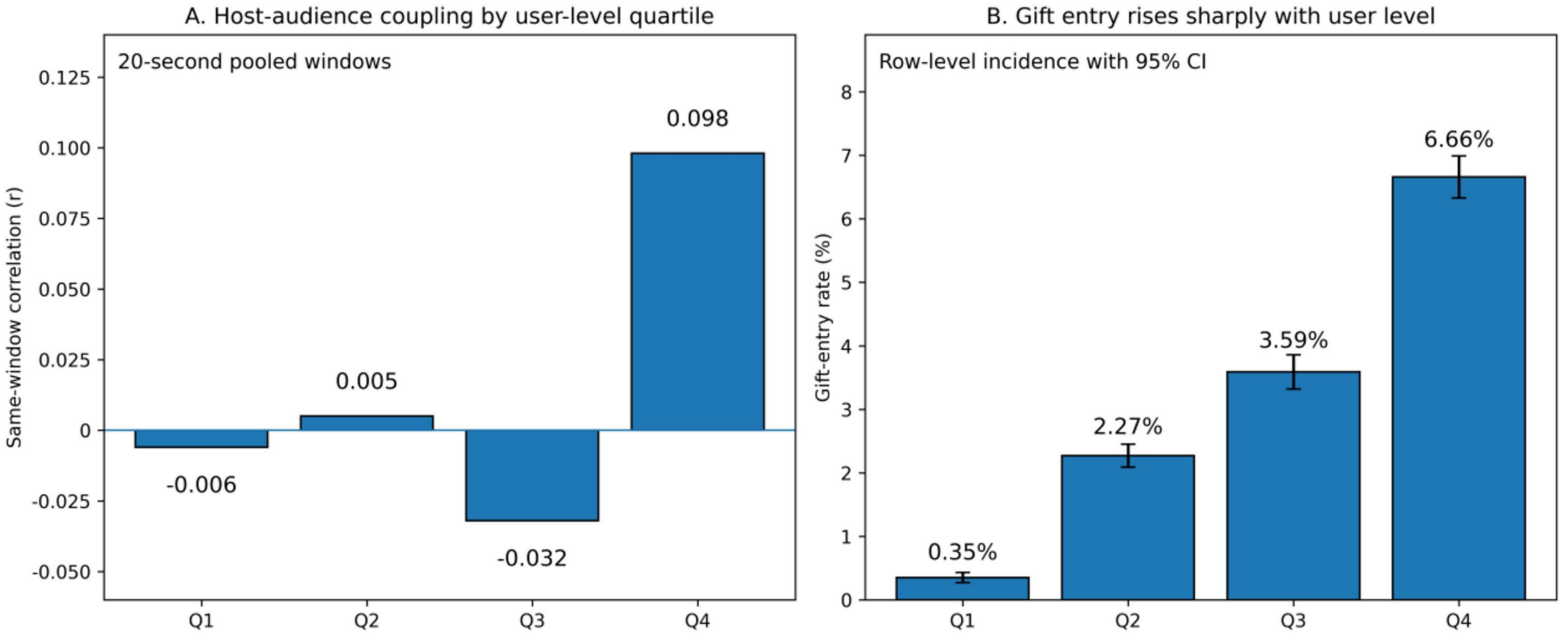
Platform stratification and visible participation. Panel **(A)** shows host-audience coupling by user-level quartile, whereas Panel **(B)** shows the gift-entry rate by user-level quartile.

An even more pronounced difference appears in gift-entry behavior. As shown in Figure 5, gift-entry rates rise monotonically from Q1 to Q4, reaching 0.35, 2.27, 3.59, and 6.66%, respectively. This gradient pattern is itself revealing: the higher the user level, the more likely viewers are to enter the gifting layer. The gift-entry rate in the highest quartile (Q4) is substantially higher than that in the lowest quartile (Q1), corresponding to an odds ratio of OR = 20.05 (95% CI [15.95, 25.20], *p* < 0.001). This gap points to more than a simple difference in overall activity. Rather, it suggests that when exposed to the same host emotional signals and race progression, higher-level users are more likely to convert shared attention into publicly visible action.

Within the 20-s response-search window, high-level users also show slightly shorter average response latency. In the pooled sample, the average response latency is 0.928 s for high-level users and 0.988 s for low-level users (*p* = 0.045). On Day 1, the corresponding values are 0.740 s for high-level users and 0.774 s for low-level users (*p* = 0.043). A similar direction is also observed on Day 2. Because the magnitude of this temporal difference is small, and because user level may also reflect prior platform activity, platform tenure, consumption tendency, fan identity, and repeated participation, this result should be treated only as auxiliary evidence of stratified participation differences. It should not be interpreted as evidence that platform level directly causes faster emotional response. Overall, the more stable manifestations of level differences remain the clearer visible coupling and higher probability of gift entry among high-level users.

### 4.5 The amplifying effect of emotional alignment on virtual consumption

**Figure 6:**
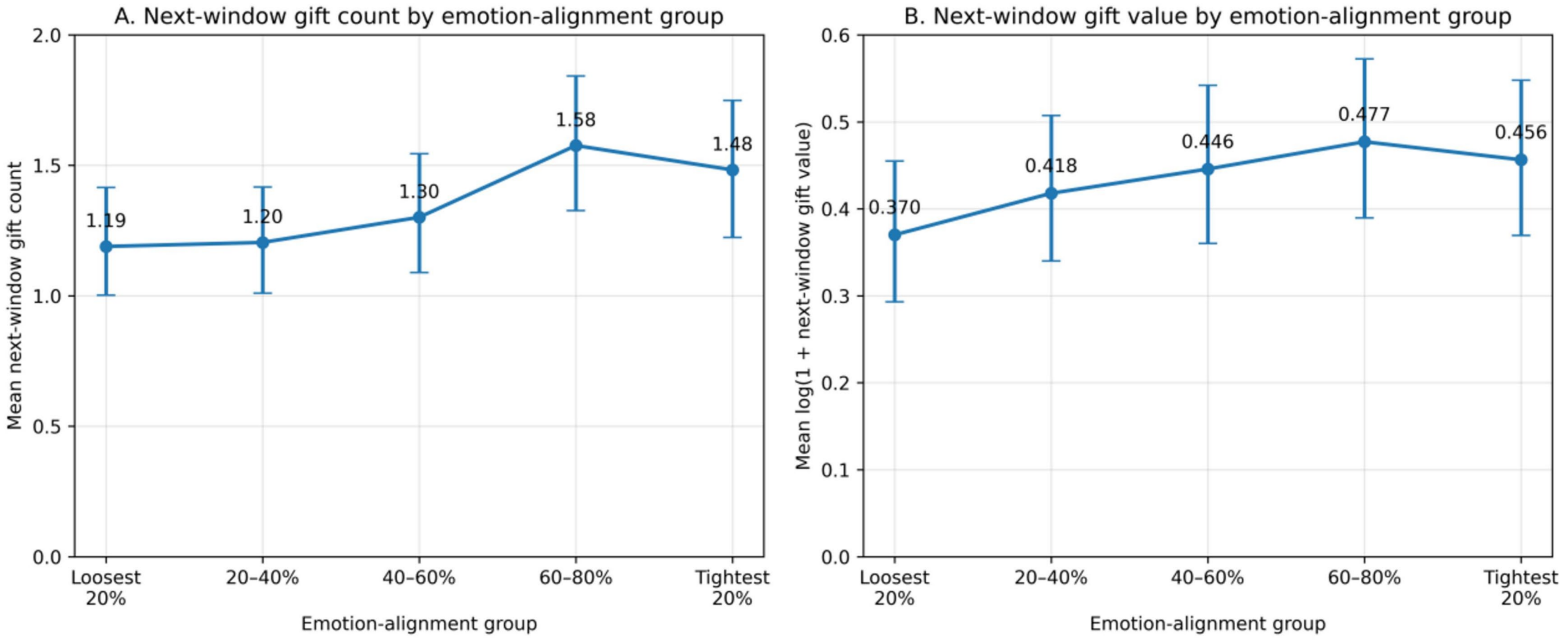
Emotional alignment and the behavior of presenting gifts in the next window. Panel **(A)** shows next-window gift count by emotion-alignment group, whereas Panel **(B)** shows next-window gift value by emotion-alignment group.

When the host and the audience are examined within the same 20-s time window, emotional alignment shows a relatively stable positive relationship with gifting behavior in the subsequent window. In the pooled forward-looking model, the smaller the emotional distance between the host and the audience in the current window, the higher the gift quantity in the next window (*β* = 7.938, SE = 2.543, 95% CI [2.954, 12.922], *p* = 0.002). Gift value shows the same directional pattern (*β* = 2.048, SE = 0.899, 95% CI [0.286, 3.810], *p* = 0.023). Given that gift quantity is a count outcome and gift value is right-skewed with many zero-valued windows, this study treats the linear model as a baseline association model and further conducts robustness checks using count models and two-part models. Therefore, this result is better understood as a temporal association between emotional alignment and subsequent visible support, rather than as causal evidence that emotional alignment directly increases gifting. Figure 6 is used only to describe the distributional trend of gifting across emotional-alignment quintiles; the formal model continues to use emotional alignment as a continuous variable. As shown in the figure, both gift quantity and gift value are generally higher after high-alignment windows than after low-alignment windows, although the difference does not follow a perfectly linear monotonic increase.

In the day-specific results, this difference becomes even clearer. On the first race day, emotional alignment shows a significant positive effect on gift quantity in the next window (*β* = 7.01, SE = 2.67, 95% CI [1.777, 12.243], *p* = 0.009), but its effect on gift value is not yet significant (*β* = 1.14, SE = 0.99, 95% CI [−0.800, 3.080], *p* = 0.248). The second race day goes further: emotional alignment not only increases gift quantity (*β* = 13.26, SE = 4.24, 95% CI [4.950, 21.570], *p* = 0.002), but also significantly increases gift value (*β* = 5.31, SE = 1.45, 95% CI [2.468, 8.152], *p* < 0.001). That is, on Day 1, emotional alignment is reflected more in bringing gifting behavior out; by Day 2, it not only makes gifts more likely to appear, but also makes them more likely to reach a higher level of value intensity.

These results indicate that gifts are not incidental behaviors detached from emotion, but action outcomes through which emotional alignment becomes further externalized. Compared with comments, gifting carries higher cost and greater visibility, and therefore more clearly reflects the process by which emotion moves from immediate expression to actual investment. The closer host and audience emotion is within the current time window, the more likely gifts are to appear in the subsequent window, and the higher their value becomes. Taken together, the four hypotheses do not receive uniform support of the same kind. Instead, their results form a progressive structure of emotional coupling, narrative regulation, hierarchical sorting, and behavioral amplification. The main test results are reported in Table 2.

**Table 2:** Main hypothesis-testing results under the 20-s specification.

Hypothesis	Test metric	Pooled	Day 1	Day 2	Verdict
H1	Same-window correlation r	0.021	0.085 **	−0.021	Partially supported
	Peak lagged correlation r [lag]	0.054 * [40 s]	0.085 ** [0 s]	0.037 * [40 s]	
H2	Peak latency *β*	1.048	0.420	1.642	Conditionally supported
	Maintenance effect *β*	0.008	−0.003	0.019 **	
H3	High/low-level same-window r	0.096***/−0.004	0.104 **/0.063 *	−0.036/0.030	Partially supported
	Gift-entry rate (high vs. low)	6.66%/0.35%	7.38%/0.39%	6.21%/0.33%	
	OR (high vs. low)	20.05 ***	20.45 ***	20.08 ***	
	Response latency (high/low, s)	0.928/0.988 *	0.740/0.774 *	0.921/0.986 **	
H4	Gift count *β*	7.938 **	7.01 **	13.26 **	Supported
	Gift value *β*	2.048 *	1.14	5.31 ***	

Values reported in the table are test statistics; * *p* < 0.05, ** *p* < 0.01, *** *p* < 0.001. Unstarred statistics are not significant. In the response-latency row, asterisks indicate the significance of the difference between the high-level and low-level groups, rather than the significance of either single value itself. In H3, the gift-entry-rate row reports descriptive proportions; the statistical significance of the high-vs.-low difference is indicated in the OR row.

## 5 Discussion and conclusion

This study uses the Chinese livestream of the Hakone Ekiden as an observational case to examine the temporal associations among host narrative, audience affective response, and subsequent visible participation in cross-cultural sports livestreaming. Overall, host and audience emotions do not show strong synchrony throughout the entire livestream. Instead, they exhibit weak and local temporal coupling in certain time windows. Day 1 is closer to weak near-immediate coupling, whereas Day 2 more clearly shows a short-lag structure in which host commentary emotion precedes and audience comment emotion follows. A2 cultural-translation narrative does not consistently shorten audience response latency, but on Day 2 it is associated with longer post-event emotional maintenance. This suggests that its role may not lie in igniting emotion more quickly, but in providing audiences with an interpretive frame that allows emotion to remain for longer. High-level users show clearer visible coupling and a higher probability of gift entry, but this pattern is intertwined with their prior platform participation and consumption tendency; therefore, it cannot be simply interpreted as a causal effect of user level itself. The results for gifting behavior further show that tighter emotional alignment between the host and the audience is associated with higher gift quantity and gift value in the next window. Taken together, digital emotional resonance in this case should not be understood as simple immediate synchrony. Rather, it is an observational process that gradually unfolds through narrative interpretation, platform visibility, and subsequent visible participation.

### 5.1 Theoretical implications

First, this study moves digital emotional resonance beyond the narrow assumption that it must occur at the same moment. Existing research has long recognized emotional transmission in livestream interaction, yet empirical analysis still tends to reduce the question of resonance to whether zero-lag synchrony is present (Zhao and Chung, 2025). The findings here suggest that such an understanding is insufficient. In the Chinese rebroadcast of the Hakone Ekiden, resonance between hosts and audiences is not absent; rather, it does not always take the same temporal form. The first race day is closer to weak immediate coupling, whereas the second race day is better understood as a structure in which hosts first organize the meaning and rhythm of the race and audience emotion follows only afterward. Under this view, lag is no longer merely a statistical displacement, but part of resonance itself. Put differently, emotion in the livestream room does not simply arise simultaneously. It takes shape gradually through the ongoing interaction of narrative progression, audience uptake, and collective response. This matters theoretically because it recasts the question from whether synchrony exists to how coupling is formed, and shifts emotional resonance from a static diagnostic category back into a dynamic processual problem.

Second, this study places the host back at the center of cross-cultural sports communication. Existing research has already shown that emotion in livestream rooms is not merely an attachment to content, but is continually amplified, extended, and reproduced through interaction (Li, 2025; Liu et al., 2025). Yet in the specific context of cross-cultural sports livestreaming, there has been little in-depth empirical analysis of how hosts participate in this process. The findings here suggest that the role of A2 does not lie in immediately pushing audience emotion upward once it appears. Rather, after the host performs a culturally translational narrative, A2 helps keep audience emotion within an already interpreted and framed field of meaning for a longer period. This difference is not very visible on the first race day, but becomes much clearer on the second. Put differently, the importance of culturally translational narrative in cross-cultural rebroadcasting lies less in igniting emotion more quickly than in sustaining it for longer. If hosts merely report race developments, audiences may know what has happened; but when hosts further organize sectional significance, competitive dynamics, athlete identity, and strategic pressure into an intelligible narrative frame, audiences are more likely to enter a structure of meaning through which they can understand why a given segment should be viewed in a particular way. For this reason, narrative mediation is treated here not as a background condition, but as a key mechanism through which emotional resonance is entered. A2, moreover, is unlikely to be a fully homogeneous category. The current results suggest that culturally translational narrative may contain several distinct themes, some of which may be more effective than others in prolonging subsequent emotion or in altering the rhythm through which audiences enter emotional resonance (Yao, 2025).

Third, the results of H3 and H4 further show that emotional flow, opportunities for participation, and visible positions in the livestream room are not evenly distributed, but always carry clear characteristics of platform stratification. Although everyone is in the same livestream room, not everyone is seen, drawn in, or converts emotion into action in the same way (Zhou, 2025). Clearer same-window coupling among high-level users, a higher probability of gift entry, and the stable prediction of subsequent gift quantity and gift value by emotional alignment all show that emotion in the livestream room is not evenly distributed and circulated. It is both filtered by platform structure and further amplified through the gifting mechanism. In this sense, the importance of high-level users lies not only in the fact that they respond faster, but also in the fact that they are more easily seen and more likely to convert emotion into public action. The meaning of gifts is not only the payment behavior itself; it is the visible result after emotional alignment is transformed into the behavioral layer. Once host-audience emotion becomes more tightly aligned, the platform provides a ready-made channel through which this short-term consistency is made public, monetized, and transformed again into a signal visible to the entire livestream room (Xu et al., 2025). In this way, narrative, emotion, status, and consumption are no longer several parallel parts, but continuous links in the same mechanism chain.

### 5.2 Practical implications

From a practical standpoint, these findings suggest that commentary in cross-cultural sports livestreaming does not become more effective simply by becoming louder, busier, or more emotionally charged, nor does mechanically spreading culturally translational content across an entire broadcast necessarily generate stronger interaction. A more effective strategy is to position A2 narrative at those key moments that genuinely require explanation, such as sectional significance, school traditions, athlete identity, competitive dynamics, and strategic change—points that cross-cultural viewers may not be able to read immediately. For cross-cultural audiences, emotion often does not come first and explanation later; rather, explanation is what enables entry into emotion (Dang-Van et al., 2026). The task of commentary, then, is not merely to make the event sound lively, but to make it intelligible, sustainable, and worth continuing to watch (Li et al., 2025a). This is especially important in cross-cultural sports rebroadcasting, where viewers are not encountering an event that is already familiar to them. In this context, the host’s role is to turn an unfamiliar sporting event into a public event that audiences can enter and stay with.

For platforms and operators, the findings also provide more concrete implications, although they should still be interpreted cautiously. High-emotional-alignment windows do not necessarily lead to gift conversion, but they often indicate that more concentrated emotional attention has already formed in the livestream room. The visible participation of high-level users in these windows also suggests that platforms should not focus only on overall popularity or traffic intensity. They should also observe whether emotion is becoming more concentrated and which users are pushing this emotion toward a more public layer of interaction. In other words, the key to platform operation is not merely to create a livelier interface, but to connect commentary explanation, audience response, and visible participation more carefully at critical narrative moments. Similarly, research on information push in e-commerce also shows that platform information is not a neutral reminder tool; rather, its goal fit, cognitive load, and emotional arousal jointly shape users’ purchase judgments and subsequent behavior (Gary, 2025). Augmented-reality retail research further suggests that digital media can influence consumers’ experience evaluations and behavioral intentions through information richness, immersion, interaction satisfaction, and anticipated emotions (Gary et al., 2025; Söderström et al., 2024). This study does not argue that sports livestreaming, information push, and AR retail operate through the same mechanism. Instead, it uses these adjacent digital settings to suggest that, in digital society, consumption action is often not triggered by emotion alone, but gradually forms as a visible outcome jointly organized by technological media, interpretive frames, and platform mechanisms.

### 5.3 Limitations and future research

This study still has several limitations, which also define the interpretive boundaries of its findings. First, the analysis is based on one Chinese-language Douyin livestream room, two race days, and one Japanese endurance-sport event. The findings are therefore more appropriately understood as an observational case with cross-cultural communication characteristics, rather than as results that can be directly generalized to all sports livestreaming contexts. Second, sentiment measurement in this study relies primarily on textual data. Multimodal cues such as video switching, subtitle rhythm, and platform interface prompts have not yet been systematically incorporated. In addition, the transfer from Weibo-based training data to Douyin sports livestream comments may introduce measurement error. Although this study reports validation metrics from the source corpus, the current livestream corpus still lacks a larger-scale independent gold-standard manual validation. Third, the 20-s window is the main specification chosen as a compromise between temporal sensitivity and measurement stability. However, livestreaming interaction itself unfolds across multiple rhythms, and no single temporal granularity can fully capture immediate response, short-lag coupling, and subsequent emotional maintenance at the same time.

Future research can proceed along several clearer lines. First, the internal thematic structure of A2 can be specified in greater detail. The current findings already suggest that A2 is not a fully homogeneous category. Culturally translational narrative likely contains several distinct themes, some of which may be more effective than others in prolonging subsequent audience emotion or altering the lag structure through which audiences enter emotional resonance. Second, future work should continue to advance multimodal analysis by integrating text, audio, visuals, and interface prompts within a single analytical framework, rather than compressing the emotional dynamics of the livestream room into the textual layer alone. Third, this mechanism chain can be tested across a wider range of settings, including other cross-cultural sports broadcasts, domestic cultural-tourism livestreams, and even product-selling livestreams, because all of these contexts confront a similar question: when viewers encounter an object that is not naturally familiar, how does narrative explanation organize emotion, and how does it gradually push that emotion toward visible action? Fourth, future research can further examine how higher-level users, gift combos, fan badges, and repeated participation reshape the visible hierarchy of resonance, rather than merely altering aggregate gift volume. Put differently, what matters most going forward is not simply whether additional significant results can be found, but how this mechanism chain of narrative, emotion, and interaction/consumption can be unpacked more finely and examined more deeply.

## Data Availability

The datasets presented in this study can be found in online repositories. The names of the repository/repositories and accession number(s) can be found at: https://osf.io/7gk9u/overview?view_only=66cabdbacc434d2f9c31a307e627ad81.
